# Comprehensive Analysis of *StSRO* Gene Family and Its Expression in Response to Different Abiotic Stresses in Potato

**DOI:** 10.3390/ijms232113518

**Published:** 2022-11-04

**Authors:** Yanming Ma, Xiangyan Zhou, Ziliang Liu, Bing Wu

**Affiliations:** 1 College of Life Science and Technology, Gansu Agricultural University, Lanzhou 730070, China; 2 Gansu Provincial Key Laboratory of Aridland Crop Science, Gansu Agricultural University, Lanzhou 730070, China

**Keywords:** potato, *SRO* genes, bioinformatics, abiotic stress, gene expression

## Abstract

As a highly conserved family of plant-specific proteins, SIMILAR-TO-RCD-ONE (SROs) play an essential role in plant growth, development and response to abiotic stresses. In this study, six *StSRO* genes were identified by searching the PARP, RST and WWE domains based on the genome-wide data of potato database DM v6.1, and they were named *StSRO1*–*6* according to their locations on chromosomes. *StSRO* genes were comprehensively analyzed using bioinformatics methods. The results showed that six *StSRO* genes were irregularly distributed on five chromosomes. Phylogenetic analysis showed that 30 *SRO* genes of four species were distributed in three groups, while *StSRO* genes were distributed in groups II and III. The promoter sequence of *StSRO* genes contained many cis-acting elements related to hormones and stress responses. In addition, the expression level of *StSRO* genes in different tissues of doubled monoploid (DM) potato, as well as under salt, drought stresses and hormone treatments, was analyzed by RNA-seq data from the online database and quantitative real-time polymerase chain reaction (qRT-PCR) analysis. Furthermore, the expression level of *StSRO* genes was analyzed by transcriptome analysis under mild, moderate and severe salt stress. It was concluded that *StSRO* genes could respond to different abiotic conditions, but their expression level was significantly different. This study lays a foundation for further studies on the biological functions of the *StSRO* gene family.

## 1. Introduction

When plants are subjected to the stress of abiotic and biotic environments, they can usually adapt to changing and complex environments by inducing the expression of stress genes [1,2,3]. Many members of the plants’ gene families were involved in plant-specific development and a series of stresses [4,5]. For example, multiple organellar RNA editing factor (*MORF)* genes regulate the plant–pathogen interaction by controlling the extent of RNA editing; growth regulatory factors (*GRF)* genes show different expression patterns in different tissues or under drought and salt stress; *MaGRAS* plays an important role in response to abscisic acid (ABA) and abiotic stress [5,6,7]. The SIMILAR-TO-RCD-ONE (*SRO*) plays a vital role in plants coping with abiotic and biotic stresses and participating in growth and development [4,8,9]. Among the plant-specific TF families, the SRO proteins were identified as a plant-specific small protein family, which played a significant role in the process of plant growth, development and coping with stresses, such as heavy metals, salt, cold and drought [1,4,9,10]. The plant SRO proteins usually contain highly conserved poly ADP ribose polymerase (PF00644; PARP) catalytic center and the C-terminal RST (RCD-SRO-TAF4; PF12174) domain. Additionally, some plant SRO proteins may possess the domain of N-terminal WWE (PF02825), which may have functions such as transcriptional regulation and modification [11,12].

The *SRO* gene family exists in all terrestrial plant genomes, and its composition varies greatly among different species. The SRO protein is a highly conserved plant-specific protein family [13,14]. Previous studies showed that although SRO proteins were usually not regulated by the transcription level, they can interact with transcription factors through the C-terminal RST domain [3,4,11]. The catalytic core of the PARP domain is the most conservative feature of the *SRO* gene family. Still, the biochemical analysis of *AtRCD1* and bioinformatics analysis of PARP domain folding structure showed that the *SRO* does not possess ADP-ribosyl transferase activity. PARP connected a single ADP ribose unit to a protein and catalyzed the extension and branching of long poly ADP ribose chains [3,13,15]. PARP plays a critical role in chromatin remodeling, telomere stability, DNA repair, transcription and cell death. Therefore, the SRO plays an essential part in plants, and it may be related to the regulation of transcription factors and the formation of complexes [3,4,13]. A total of six *SRO* homologous genes were identified and obtained from Arabidopsis, and the six members were *AtSRO1*–*5* and *AtRCD1* [16,17]. Studies have shown that Arabidopsis Clone eight-one (*CEO1*) could enhance the ability of yeast activator proteins 1 (Yapl) mutant and wild-type yeast to resist oxidative stress, which showed that *AtCEO1* played a vital part in biological oxidative stress response [18,19]. In subsequent studies, it was found that the *AtCEO1* gene belongs to the plant’s specific *SRO* gene family, and its mutation may lead to the rapid death of plant cells, so *AtCEO1* was also called *AtRCD1* (Radical-induced cell death 1) [17,18,19,20,21]. *AtRCD1* was the first member of the *SRO* gene family identified in Arabidopsis. Generally, *AtRCD1* could be involved in drought stress mediated by the ABA signaling pathway through interaction with transcription factors in the nucleus. Additionally, it could also regulate plant growth and development by participating in some hormone signaling pathways, such as ABA, methyl jasmonate (MeJA) and ethylene (ETH) [4,13,18]. *AtRCD1* and *AtSRO1* are highly homologous, and both of them have three domains. With the common PARP and RST conserved domains, they also have an N-terminal WWE domain and belong to type A, while *AtSRO2*–*5* lack the WWE domain and only contain two domains of PARP and RST, which belong to type B [19,20,21].

Potato (*Solanum tuberosum* L.) originated from the Andean regions of Peru and Bolivia and is considered to be one of the most important food crops worldwide [22,23]. However, the yield and quality of potato are severely affected by various abiotic stresses, particularly drought and salt stresses [23,24].

*SRO* genes have been reported in many plants. The *SRO* gene family may be a transcriptional regulator in plant response to multiple abiotic stresses [1,4,9,10,12,13,14,16]. However, little is known about the gene structure characteristics and functions of the *StSRO* gene family, especially their functions under stresses in potatoes. Therefore, it is crucial to understand the gene structure of *StSRO* and its function under abiotic stress. In this study, the physical, chemical and structural characteristics of the *StSRO* gene family were identified using bioinformatics methods. Their conserved motifs, chromosome distribution, evolutionary relationship, cis-acting elements and protein network interaction were studied and validated. The expression patterns of *StSRO* genes in different tissues of potato were analyzed. The expression levels of *StSRO* genes under exogenous ABA treatment, salt and drought stress treatment were analyzed by RNA-seq data from the online database and qRT-PCR, and their expression levels under salt stress were also analyzed by transcriptomics. The results lay a foundation for further study of the structure or functions of *StSRO* genes.

## 2. Results

### 2.1. Identification of SRO Genes in Potato

SRO protein coding genes were determined, and a total of six *StSRO* genes were obtained by searching the PARP, RST and WWE domains in the potato database DM v6.1. All *StSRO* genes were named *StSRO1*–*6* according to their distribution on the chromosome. Additionally, ten transcripts of the *StSRO* gene family were identified in the potato database DM v4.03/4.04, which correspond to the transcripts of the *StSRO* gene family in the potato database DM v6.1 [25,26]. In this study, six *StSRO* genes identified from the genome of potato database DM v6.1 were used as the research object. As listed in Table 1, six *StSRO* genes have at least one and up to three transcripts. The six *StSRO* genes were divided into two types according to whether their N-terminal contained the WWE domain or not. Only the *StSRO6* gene has three domains: PARP, RST and WWE. While *StSRO1*–*5* lacked the WWE domain, they included only two domains: PARP and RST. 

As shown in Table 2, the lengths of the six StSRO proteins identified were between 316 and 594 AA; the molecular weight (MW) of StSRO proteins varied between 33,719.99 and 67,469.25 KD. The isoelectric point (pI) varied significantly from 5.97 to 9.16; among them, the pI of StSRO2, StSRO5 and StSRO6 were below 7.0, while the pI of StSRO1, StSRO3 and StSRO4 were higher than 7.0. Therefore, it can be inferred that StSRO2, StSRO5 and StSRO6 were acidic proteins, and StSRO1, StSRO3 and StSRO4 were essential proteins. Since the grand average of hydropathicity (GRAVY) values predicted for StSRO proteins ranging from −0.446 to −0.234 were all negative values, it can be inferred that they were hydrophilic proteins. The instability index (II) of StSRO proteins ranged from 38.53 to 52.78 (only one below 40), that is, most StSRO proteins were unstable. The aliphatic index (AI) of StSRO proteins was between 68.46 and 88.2, and StSRO1 and StSRO4 had the lowest and highest aliphatic indices, respectively. The subcellular localization (SL) predicted that most StSRO proteins were located in the nucleus or cytoplasm, where the number of subcellular localizations of StSRO2 and StSRO3 proteins was the highest, while the number of subcellular localizations of StSRO5 was the lowest.

### 2.2. Gene Structure and Conserved Motifs of SRO Proteins in Potato

**Figure 1:**
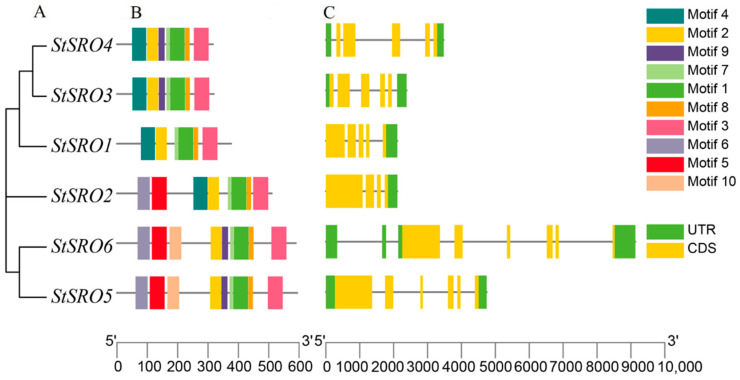
Phylogenetic relationship, conserved motifs and gene structure of *StSRO* genes. (**A**) Phylogenetic relationship of *StSRO* genes, (**B**) the ten conserved motifs of *StSRO* genes are displayed in different colors, (**C**) the exon-intron structure. The coding sequence (CDS) and untranslated region (UTR) are displayed in different colors, and the lines between the boxes represent introns.

The evolution tree of *StSRO* gene family members was constructed separately and predicted by TBtools software. Ten conserved motifs of *StSRO* genes were identified (Figure 1). Generally, the difference in protein function is caused by the different protein motifs [21]. Therefore, the conservative motif analysis of *StSRO* genes showed that motifs 1, 2, 3, 7 and 8 were highly conserved and existed in the amino acid sequence of each *StSRO* gene, so it was speculated that these five motifs might play an important role in the *StSRO* gene family. *StSRO1*–*4* contained motif 4, while motif 5 and motif 6 existed in *StSRO2*, *StSRO5* and *StSRO6*; motif 9 existed in *StSRO3*–*StSRO6*; motif 10 only existed in *StSRO5*–*6*.

The exon-intron structure is not only an essential evolutionary feature of genes but also a critical clue to the diversification of gene functions [1]. Therefore, the exon-intron structure of *StSRO* genes was analyzed. The results showed that *StSRO* genes with a close genetic relationship have a similar exon-intron structure, and six members of the *StSRO* genes had three to seven introns with different lengths, of which *StSRO2* had the lowest number of introns (three), while *StSR06* had the highest number of introns (seven).

### 2.3. Chromosomal Mapping and Secondary Structure Analysis of StSRO Genes in Potato

**Figure 2:**
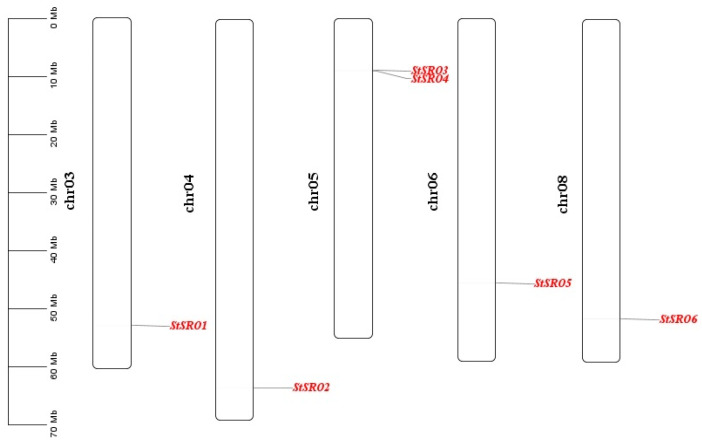
The chromosomal mapping analysis of *StSRO* gene family in potato.

The chromosome mapping analysis of *StSRO* genes showed that six *StSRO* genes were randomly distributed on five chromosomes (Figure 2). Among them, *StSRO3* and *StSRO4* were distributed on chromosome 5; *StSRO1*, *StSRO2*, *StSRO5* and *StSRO6* were distributed on chromosome 3, 4, 6, 8. Among these six *StSRO* genes, *StSRO3* and *StSRO4* were distributed adjacent on chromosome 5, which may be caused by tandem duplicated events of genes in chromosome regions. 

**Figure 3:**
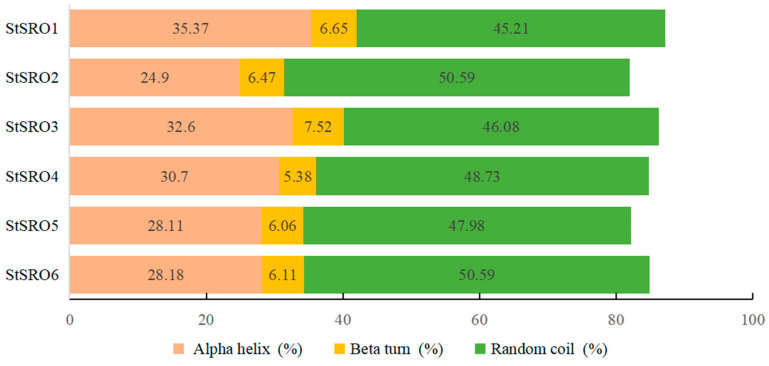
Secondary structure prediction of StSRO proteins.

There were four main secondary structures of proteins, including α-helix, β-sheet, β-turn and random curl. Because the MW of the protein was relatively large, this may lead to different forms of secondary structure in different peptide segments of a protein [27]. According to the predicted secondary structure of six StSRO proteins (Figure 3), the secondary structures of StSRO proteins were mainly α-helix and random curl structures; the β-turn structure accounted for a small proportion. The random curl structures of StSRO2 and StSRO6 accounted for 50.59%, and the α-helix structure represented 24.9% and 28.18%, respectively.

### 2.4. Synteny Analysis of SRO Gene Family

**Figure 4:**
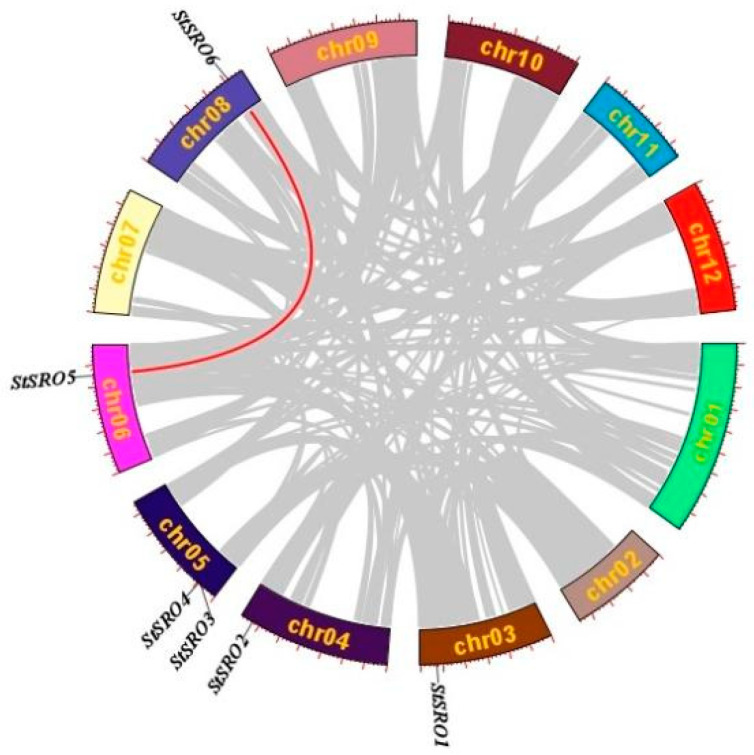
Synteny analysis and chromosome location of *StSRO* genes in potato. Red lines represent the syntenic relationships of *StSRO* genes.

In order to identify the duplications of *StSRO* genes, the segmental duplication of the potato genome during evolution was analyzed. As shown in Figure 4, there was one segmental duplication event (*StSRO5*/*StSRO6*) between different chromosomes. The Ka/Ks ratio between duplicate gene pairs was calculated, and the Ka/Ks value was less than 1 (Table 3), which showed that they had undergone purification selection in the process of evolution.

**Figure 5:**
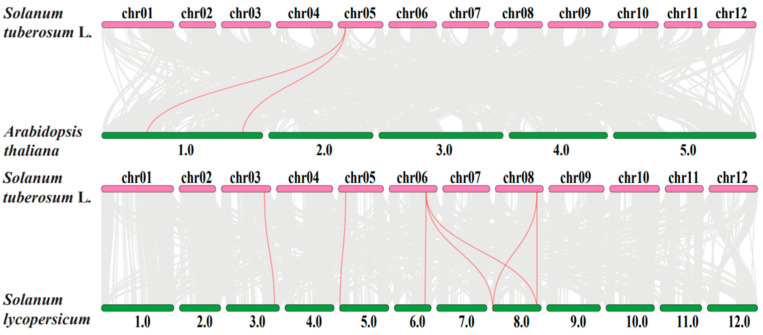
Synteny relationships of *SRO* genes between potato and Arabidopsis, tomato. Gray lines represent collinear blocks between potato and Arabidopsis, tomato genomes. Red lines represent syntenic *SRO* gene pairs among potato and Arabidopsis, tomato.

It has been shown that the *SROs* gene has similar expression patterns in response to certain stresses in potato and tomato, such as salt stress [28]. Therefore, to explore the orthologous relationships of *SRO* genes among potato, tomato and Arabidopsis, the collinearity relationship of them was analyzed by TBtools with MCScanX. The results showed two orthologous gene pairs between potato and Arabidopsis (Figure 5), and seven orthologous gene pairs were identified between potato and tomato. Therefore, the synteny relationships among potato, Arabidopsis and tomato indicated that potato had a closer evolutionary relationship with tomato.

### 2.5. Phylogenetic Tree Analysis of SRO Genes

**Figure 6:**
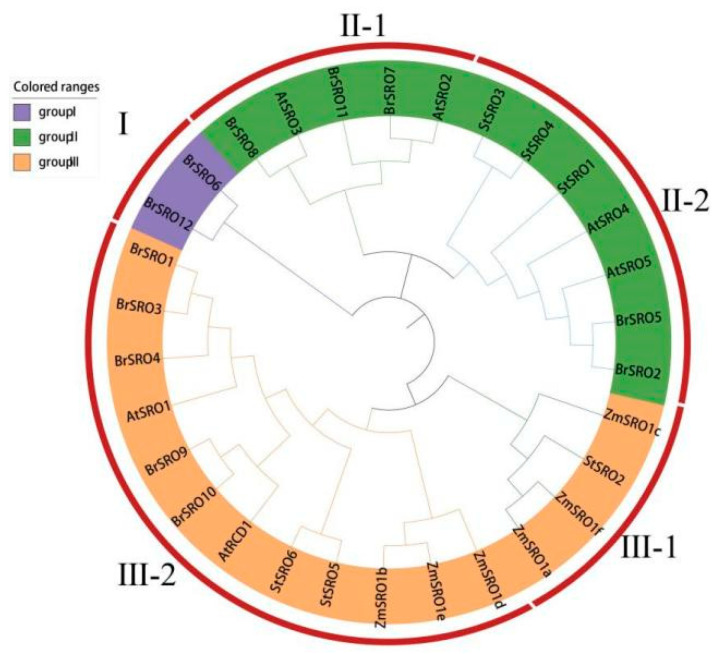
Phylogenetic tree analysis of *SROs* gene family in potato (St), *Arabidopsis thaliana* (At), *Brassica pekinensis* (Br) and *Zea mays* (Zm).

To understand the phylogenetic relationship of the *StSRO* gene family, the protein sequences of 30 *SRO* genes of potato, Arabidopsis, *Brassica pekinensis* and *Zea mays* were compared by ClustalW. Then, the phylogenetic trees of four species were constructed (Figure 6). According to the results of evolutionary branches, *SRO* genes could be divided into three groups. Group II and group III have two subgroups, respectively, in which *StSRO1*, *StSRO3* and *StSRO4* were clustered in subgroup II-2; *StSRO2* was clustered in subgroup III-1; *StSRO5* and *StSRO6* were clustered in subgroup III-2. In addition, the *SRO* genes in group I were only from the *Brassica pekinensis*; the *SRO* genes in group II were found in potato, *Brassica pekinensis* and Arabidopsis; and the *SRO* genes included in group III were found in *Brassica pekinensis*, potato, Arabidopsis and *Zea mays*, which showed that potato was closely related to *Brassica pekinensis* and Arabidopsis but far from *Zea mays* in evolutionary relationships.

### 2.6. Analysis of Cis-Acting Elements of SRO Gene Family in Potato

**Figure 7:**
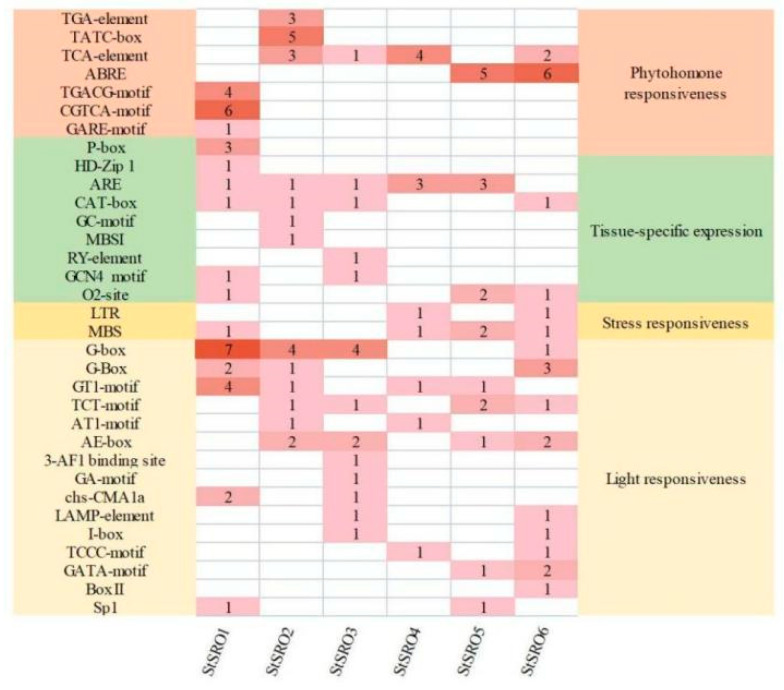
Analysis of cis-elements in the promoter region of *StSRO* genes. The number in the box represents the number of cis-acting elements.

The cis-acting elements of a promoter are a critical binding region of transcription initiation factors and play a vital role in gene expression regulation [29]. To analyze the biological functions of *StSRO* genes, 33 main homeopathic regulatory elements were screened from six *StSRO* genes (Figure 7). They were divided into four categories: seven phytohormone responsiveness elements, nine tissue-specific expression elements, two stress responsiveness elements and fifteen light responsiveness elements. Among them, the light responsiveness elements had the highest number, and the numbers of G-box, G-Box and GT1 motif cis-elements were the highest in the gene family; the stress responsiveness elements had the lowest number, and there were no stress responsiveness elements in *StSRO2* and *StSRO5*. In terms of tissue-specific expression and phytohormone responsiveness, each member of the *StSRO* genes contained corresponding cis-acting elements, and some cis-acting elements existed only in one gene, such as the RY-element, TATC-box, GARE-motif, TGA-element, P-box and GC motif, and so on. These results showed that *StSRO* genes participated in the growth and development of potato plants by responding to different cis-acting elements.

### 2.7. Construction of SRO Protein Interaction Network and Functional Annotation in Potato

**Figure 8:**
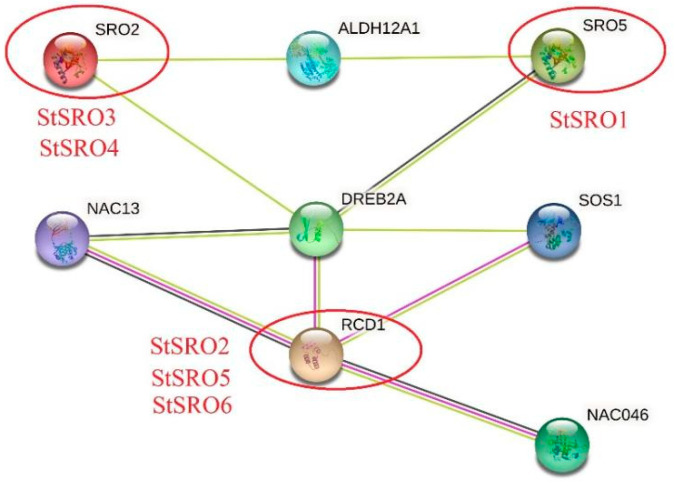
StSRO protein–protein clustering interaction network diagram.

The protein–protein clustering interaction network is composed of proteins through their interaction with each other, which is involved in various aspects of life processes, such as biological signal transmission, gene expression regulation, energy and material metabolism and cell cycle regulation [30,31,32,33]. The clustering of proteins can analyze the interaction between proteins in biological systems, which is of great significance for understanding how proteins work in a biosystem, the reaction mechanisms of biological signals and energy metabolism under special physiological conditions and the functional relationship between proteins [30,32,33,34]. Connecting unknown functional proteins to a protein–protein interaction network through protein network interaction is helpful to further understand the rich protein biological functions through protein network interaction and the dynamic network regulation between various biomolecules in cells [34,35]. This study was based on the model plant Arabidopsis to predict the physical and chemical properties of StSRO proteins and their potential interacting proteins related to their functions (Figure 8). Three functional SROs and five potential interacting proteins directly associated with the StSRO proteins were identified. They were SRO2, SRO5, RCD1 and ALDH12AL, NAC13, NAC046, DREB2A, SOS1. SRO2 and SRO5 have similar a structure and function, encoding a protein similar to RCD1 but without the WWE domain. The protein had a PARP signature upstream of the C-terminal protein interaction domain. Additionally, the PARP signature may bind NAD^+^ and attach the ADP-ribose-moiety from NAD^+^ to the target molecule. Its existence proved that SRO2 and SRO5 proteins played a role in ADP ribosylation [4,8]. RCD1 encoded a protein belonging to the (ADP-ribosyl) transferase domain-containing subfamily of the WWE protein–protein interaction domain protein family. Superoxide radicals were necessary and sufficient to propagate cell death or lesion formation in RCD1 mutants. RCD1 was localized in the nucleus without stress treatment. However, RCD1 was found not only in the nucleus but also in the cytoplasm under high salt or oxidative stress [4,8]. Therefore, the functions of the six StSRO proteins were similar to the above three Arabidopsis transcription factors. StSRO3 and StSRO4 may have similar functions to SRO2; StSRO1 may have similar functions to SRO5, while StSRO2, StSRO5 and StSRO6 may have similar functions to RCD1.

### 2.8. Functional Annotation of SRO Genes in Potato

**Figure 9:**
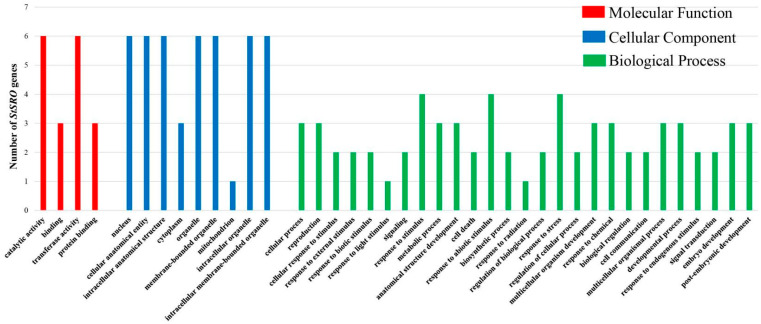
Gene ontology (GO) annotation of *StSRO* genes in potato. The results were divided into three categories:”BP”, “CC” and “MF”. The x-axis and y-axis were the GO term and the number of *StSRO* genes, respectively.

The gene ontology (GO) annotation of *StSRO* genes was carried out to demonstrate the possible functional classifications. As Figure 9 and Table 4 show, *StSRO* genes were annotated and classified into 40 functional groups in the categories of “Biological Process, BP”, “Cell Component, CC” and “Molecular Function, MF”. In terms of the MF, it primarily focused on catalytic activity (GO:0003824) and transferase activity (GO:0016740). Among the CC, the reaction mainly occurred in the nucleus (GO:0009987), cytoplasm (GO:0005737), intracellular organelle (GO:0043229) and intracellular membrane-bounded organelle (GO:0043231), which was also highly consistent with subcellular localization prediction. In BP, it mainly focused on responding to biological stimulus (GO:0050896), responding to stress (GO:0006950), responding to stimulus (GO:0050896) and so on. The GO data showed that *StSRO* genes were essential in regulating gene expression.

### 2.9. Effects of Abiotic Stresses and Hormone Treatments on Potato Plantlets In Vitro

**Figure 10:**
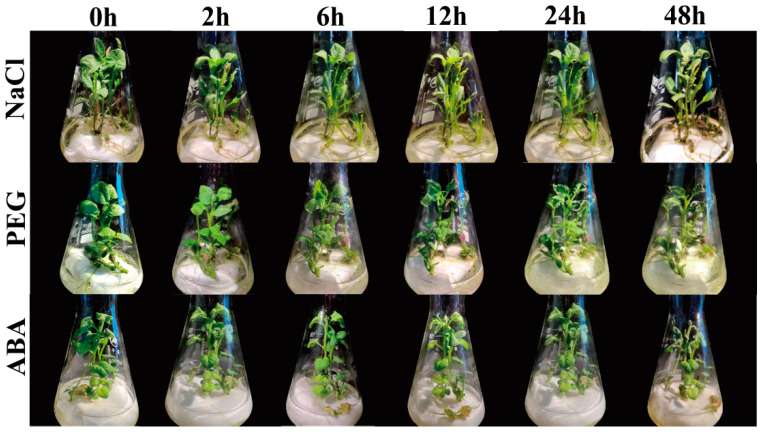
Phenotype changes in potato plantlets cultured for 30 d and treated for different durations (2 h, 6 h, 12 h, 24 h, 48 h) under salt stress (200 mM NaCl), drought stress (20% PEG-6000), hormone ABA stress (100 μM) and the control (0 h).

In order to understand the effect of salt stress, drought stress and ABA treatment on potato plantlets, this study observed the phenotype changes of in vitro potato plantlets with different treatment times (2 h, 6 h, 12 h, 24 h, 48 h) under 200 mM chloride (NaCl), 20% polyethylene glycol-6000 (PEG-6000), 100 μM ABA and the control (0 h) (Figure 10). Under salt stress, the results showed that the changes of in vitro potato seedlings were not obvious from 0 to 6 h, and the leaves appeared to wilting slightly from 12 h. The degree of wilting increased with the treatment time from 24 to 48 h. The leaves obviously withered and showed chlorosis under severe salt stress (48 h).

Under drought stress, the changes of potato plants were not significant from 0 to 6 h treatment, and the leaves showed slight wilting after 6 h. The degree of wilting increased with the treatment time from 12 to 48 h. The leaves began to show chlorosis at 24 h. The leaves appeared wilted, shrunken and browning under severe drought stress (48 h).

Under the ABA treatment, in vitro potato plants did not change significantly from 0 to 2 h treatment, and the leaves showed slight wilting after 2 h. The degree of wilting increased with the treatment time from 6 to 48 h. Chlorosis appeared on the leaves at 12 h, and the plants withered, and leaves were completely withered under severe ABA treatment (48 h).

### 2.10. Analysis of SRO Gene Expression in Different Tissues and Treatments in DM Potato

**Figure 11:**
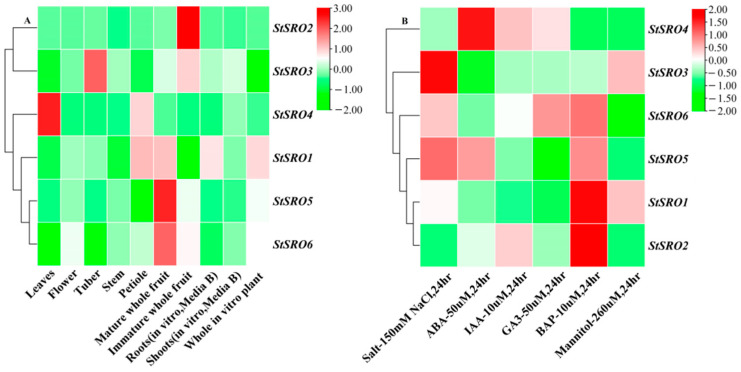
Expression profiles of *StSRO* genes in various potato organs and different treatments: (**A**) The expression profiles of *StSRO* gene family in different potato tissues, (**B**) The expression profiles of *StSRO* genes under six different treatments at 24 h. The color scale varies from green to red, indicating that the gene expression level from low to high.

The analysis of tissue-specific gene expression patterns can provide clues for the possible function of genes in development [36,37]. Therefore, the DM potato RNA sequencing data published on the online database were downloaded to analyze the changes in *StSRO* gene expression in different tissues and stresses. The results showed that (Figure 11A) the expression of *StSRO1* was higher in the petiole, mature whole fruit, roots and whole in vitro plant, while the expression was lower or absent in other tissues. The expression of *StSRO2* was only present in immature whole fruit of potato but not in other tissues. *StSRO3* was highly expressed in tubers and immature whole fruits, but its expression level was low in other tissues. The expression of *StSRO4* was higher in leaves and petiole but lower in other tissues. The expression patterns of *StSRO5* and *StSRO6* were similar, both being expressed only in the mature whole fruit, and the expression level was low or absent in other tissues. All *StSROs* expressed in flowers, stems and shoots were at a low level. Thus, most *StSRO* genes play a vital role in the growth and development of potatoes, and *StSRO* genes were highly expressed in petiole and immature fruit. Therefore, *StSRO* genes may have indispensable biological functions in potato petiole and immature whole fruit.

In addition, by analyzing the expression patterns of *StSRO* genes under six different stress treatments at 24 h (Figure 11B), *StSRO3*, *StSRO5* and *StSRO6* were highly expressed under salt treatment. *StSRO4* and *StSRO5* were upregulated under ABA treatment. *StSRO4* and *StSRO6* were highly expressed under gibberellic acid (GA3) treatment. *StSRO1*, *StSRO2*, *StSRO5* and *StSRO6* were upregulated under bone alkaline phosphatase (BAP) treatment. Under indoleacetic acid (IAA) and mannitol treatments, the expression of *StSRO* genes was low or even absent. 

### 2.11. Expression Levels of StSRO Genes under NaCl, PEG and ABA Treatments 

**Figure 12:**
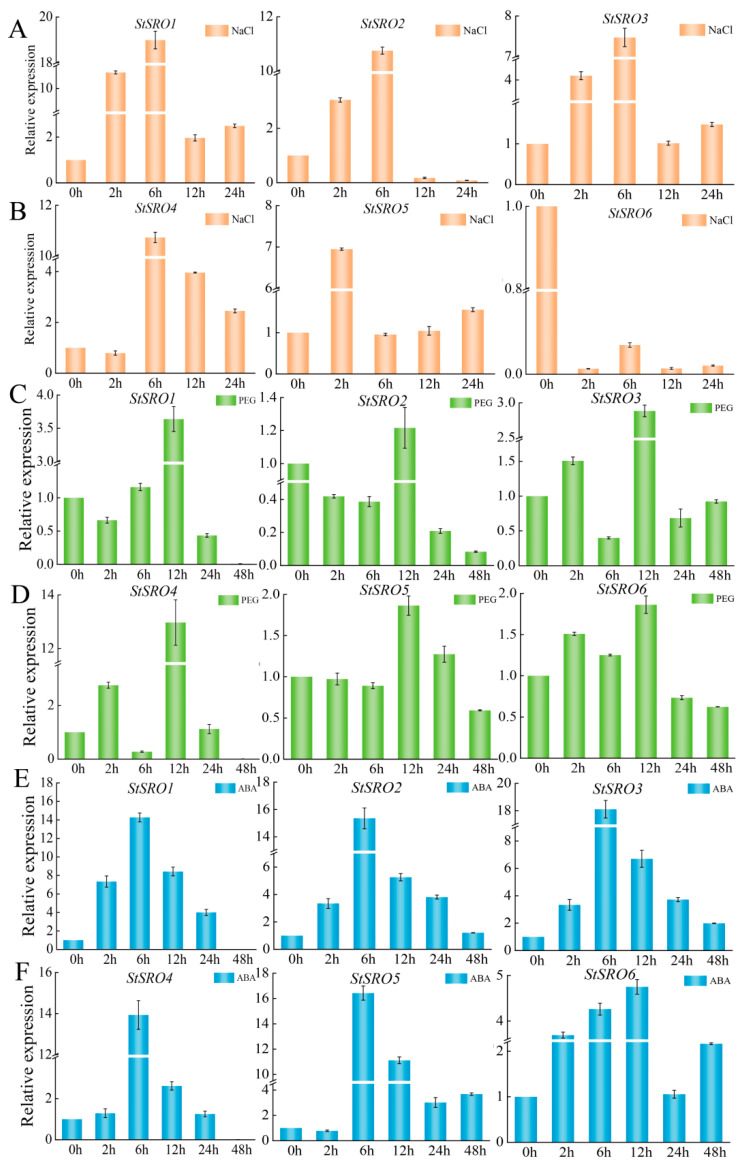
Expression analysis of *StSRO* genes under (**A**,**B**) NaCl, (**C**,**D**) PEG stress and (**E**,**F**) ABA treatment.

In addition, the expression levels of *StSRO* genes under salt, exogenous ABA treatment and drought stress were analyzed by qRT-PCR, with 0 h treatment as control (Figure 12). Under each treatment, most *StSRO* genes induced expression levels, which varied at different treatment times. Under 200 mM NaCl stress treatment, the expression levels of *StSRO1* were significantly higher than the control. *StSRO1*, *StSRO3* and *StSRO5* had the same expression pattern. However, the expression of *StSRO1* and *StSRO3* reached a peak at 6 h, while the expression of *StSRO5* peaked at 2 h. The expression levels of *StSRO2* showed a trend of increasing and then decreasing and reached a peak at 6 h. The expression of *StSRO4* showed a trend of decreasing at first, rising at 6 h and then falling. Meanwhile, the expression level of *StSRO6* was lower than that of control at four different treatment times. 

Under the 20% PEG treatment, the expression patterns of *StSRO1*, *StSRO2* and *StSRO5* were similar. *StSRO3*, *StSRO4* and *StSRO6* showed a similar expression pattern, and the expression of all *StSRO* genes peaked at 12 h. The peak expression of *StSRO1*–*6* genes was 3.5-fold, 1.2-fold, 2.8-fold, 12.9-fold, 1.9-fold and 1.9-fold that of the control, respectively.

Under exogenous 100 μM ABA, the expressions of *StSRO1*, *StSRO2*, *StSRO3*, *StSRO4* and *StSRO6* were upregulated at each treatment time. The expression levels of *StSRO1*–*5* genes peaked at 6 h. The relative expression at the peak was 14.2-fold, 15.2-fold, 18.1-fold, 13.8-fold and 16.5-fold that of the control, respectively. While the expression of *StSRO6* reached a peak at 12 h, it was 4.7-fold that of the control. Compared with the control (0 h), the *StSRO* genes, except *StSRO1* and *StSRO4,* could respond positively to severe ABA treatment (48 h). Compared with moderate ABA treatment (24 h), only *StSRO5* and *StSRO6* were upregulated. The qRT-PCR results were basically consistent with RNA-seq data from the online database.

Overall, these results indicated that *StSRO* genes could respond to salt stress, drought stress and exogenous hormone ABA treatment to different degrees, except *StSRO6,* which could not respond to salt stress. Among them, *StSRO1* could positively respond to salt stress, while *StSRO6* did not. The expression of the *StSRO* gene reached a peak at 12 h under drought stress. The expressions of *StSRO2*, *StSRO3* and *StSRO6* were significantly upregulated with different treatment times.

### 2.12. Transcriptome-Based Expression Profiling of StSRO Genes in Potato Cultivar “Zihuabai” under Salt Stress

**Figure 13:**
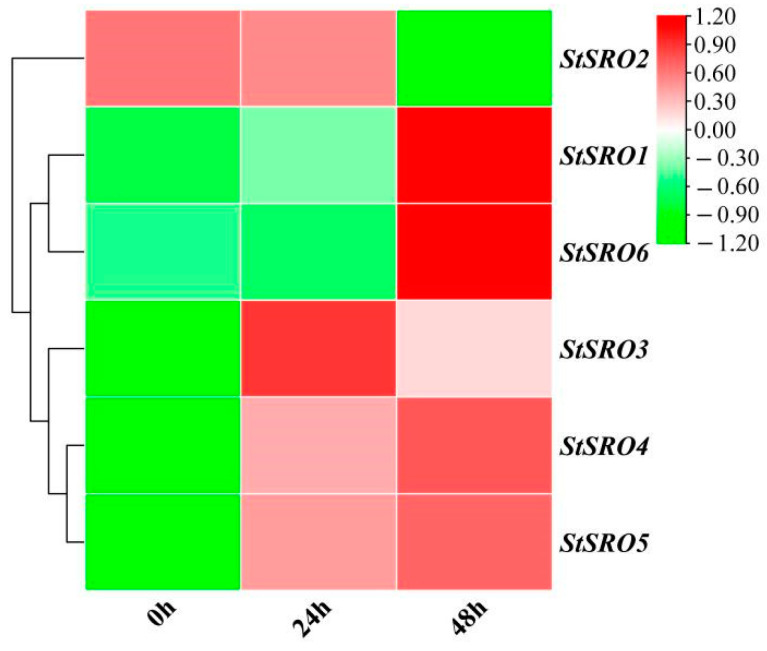
*StSRO* genes expression based on transcriptome analysis.

Furthermore, the transcriptome analysis of potatoes under salt stress was not published in our research (the transcriptome data were validated) [38], in which the FPKM values were used to draw the heatmap (Table S1 and Figure 13). The results showed that six *StSRO* genes were differentially expressed under 200 mM NaCl at different treatment times. *StSRO1* and *StSRO6* were downregulated under control (0 h) and moderate salt stress (24 h) while being highly expressed under severe salt stress (48 h). *StSRO2* was upregulated under control (0 h) and moderate salt stress (24 h) but downregulated under severe salt stress (48 h). By comparing the gene expression levels of control (0 h) and moderate salt stress (24 h), it was found that the expression levels of *StSRO2* and *StSRO6* were downregulated. In contrast, the expression levels of *StSRO1* and *StSRO3*–*5* were upregulated. The comparison of gene expression between control (0 h) and severe salt stress (48 h) showed that the expression levels of *StSRO2* were significantly downregulated, and the expressions of the other five *StSRO* genes were significantly upregulated. The comparison of gene expression between moderate (24 h) and severe (48 h) salt stress showed that the expressions of *StSRO2* and *StSRO3* were downregulated, while the expressions of *StSRO1* and *StSRO4*–*6* were upregulated. The expression levels of all *StSRO* genes under moderate salt stress (24 h) were consistent with the published RNA-seq data from the online database (Figure 11B) and our qRT-PCR results (Figure 12). Therefore, *StSRO* genes not only respond to mild and moderate salt stress (Figure 11B and Figure 13) but also notably respond to severe salt stress (Figure 13).

## 3. Discussion

As a highly conserved plant-specific protein family, the SRO protein plays a crucial role in the response of plants to abiotic and biological stresses. The SRO family exists in all terrestrial plant genomes, but its composition varies greatly among different species. The number of *SRO* gene family members identified in other species (tomato, maize, wheat, rice, Chinese cabbage, apple, banana, tea plants, upland cotton) were different, including 6, 6, 30, 5, 12, 6, 6, 9 and 12 SRO members, respectively [1,4,8,12,13,28,39,40,41]. In this study, a total of six *StSRO* genes were identified based on conserved domains. They were divided into two categories according to the presence or absence of the WWE domain: type A and type B. *StSRO6* had three domains of PARP, RST and WWE, which belonged to type A. Meanwhile, *StSRO1*–*5* had only two domains of PARP and RST, and it lacked the WWE domain, which belonged to type B. 

The length range of the six StSRO proteins was 316–594 AA; the variation range of pI was 5.97–9.16; and the MW range of the six StSRO proteins was 33,719.99–67,469.25 KD. Subcellular localization of StSRO proteins showed that most StSRO proteins mainly existed in the nucleus or cytoplasm. Chromosome analysis showed that the six *StSRO* genes were irregularly distributed on five chromosomes. The exon-intron structure provides additional information for phylogenetic analysis and plays an important role in the evolution of gene families [41,42,43,44]. By analyzing the gene structures, conserved motifs and phylogenetic relationships of *StSRO* genes, it was revealed that there were similar gene structures and conserved motifs in the same group, indicating a significant correlation between the phylogenetic relationships of *StSRO* genes and *StSRO* gene structures. At the same time, the secondary structure of StSRO proteins also predicted and found that there were α-Helix and random curl, two main secondary structures of StSRO proteins. By constructing the phylogenetic tree of potato, Arabidopsis, *Brassica pekinensis* and *Zea mays*, the results showed that potato was closely related to *Brassica pekinensis* and Arabidopsis, while potato was far from *Zea mays* in evolutionary relationships. 

Previous studies have shown that gene duplications were supposed to be one of the primary driving forces of genetic evolution, and gene duplications can occur through various mechanisms, including tandem duplications, segmental duplications and retroposition [45,46]. In the present study, the results of chromosomal localization showed the tandem duplication events of *StSRO3* and *StSRO4*. In addition to tandem duplication, we also obtained one gene pair, *StSRO5* and *StSRO6,* through genome-wide synteny analysis of the potato genome, which was a segmental duplication. From these results, it was concluded that tandem duplications and segmental duplications were involved in the potential functional diversity of *StSRO* genes and the expansion of the *StSRO* gene family.

Promoter activity plays a key role in regulating gene function [46,47]. We obtained seven phytohormone responsiveness elements, nine tissue-specific expression elements, two stress responsiveness elements and fifteen light responsiveness elements by analyzing the cis-acting elements of *StSRO* genes. Thus, *StSRO* genes can respond to plant hormones, tissue-specific expression and light responsiveness. Additionally, these results indicated that *StSRO* genes were involved in the growth and development of potatoes by responding to different cis-acting elements. The complex interactions between *StSRO* genes were detected and obtained from the protein–protein interaction network [47]. The results of the protein–protein interaction network obtained in this study suggested a possible interaction between these StSRO proteins in potato growth and development. This conclusion provides new insight into studying the regulation of potato growth and development by SROs. This study also revealed the possible role of *StSRO* genes through GO analysis. *StSRO* genes were mainly enriched in BP, CC and MF groups and played corresponding functions. This indicated that *StSRO* genes played an important role in regulating gene expression.

Generally, the expression patterns of genes were closely related to their potential functions. In Arabidopsis, *AtRCD1* and *AtSRO1* were highly expressed in young developing tissues [11]. In sesame, group I *SiSROs* were generally expressed in all tissues, while group II *SiSROs* were highly expressed in root [9]. In rice, *OsSRO1a* and *OsSRO1c* exhibited tissue- and organ-specific expression patterns [12]. The results obtained from RNA-seq data from the online database showed that *StSRO* genes were expressed in multiple tissues. *StSRO2* was highly expressed in the immature whole fruit; *StSRO3* was highly expressed in the tubers; *StSRO3* was highly expressed in the leaves; and *StSRO5* and *StSRO6* were highly expressed in the mature whole fruit. Therefore, *StSRO* genes had critical biological functions in potato leaves, tubers, immature whole fruit and mature whole fruit. 

A vast number of studies have shown that *SRO* genes can not only respond to abiotic stress but also to stress-related hormones. In this study, to verify the stress-specific expression of *StSRO* genes in the downloaded and our RNA-seq data, *StSRO* genes were confirmed by qRT-PCR in potato leaves treated with NaCl, PEG and ABA treatments. Under salt stress, qRT-PCR, our transcriptome data and online transcriptome data were basically consistent; the reason may be that the *StSRO* gene has different tolerance to salt stress caused in different potato varieties and different NaCl concentrations. Under the ABA treatment, the results of qRT-PCR were basically consistent with the online transcriptome data, but no drought stress related data were obtained from the online RNA-seq database.

The *SRO* gene family has been shown to respond to ABA treatment in many plants. The former results showed that the upregulated genes in response to ABA in tea plants were *CsRCD3* and *CsRCD4* [32]. In rice, *OsSRO1c* was obviously upregulated under ABA treatment [12]. In apples, *MdSRO4* expression was upregulated and was 14-fold that of the control under ABA treatment [13]. In bananas, *MaSRO5* and *MaSRO6* were more sensitive to ABA treatment [48]. The results of exogenous ABA treatment in this study showed that the expression of *StSRO2, StSRO3* and *StSRO6* was dramatically upregulated at different treatment times. It can be concluded that the three members were more sensitive to ABA treatment than the other members in potatoes. *StSRO2*, *StSRO3*, *StSRO5* and *StSRO6* could respond to severe ABA treatment, while *StSRO1* and *StSRO4* were not expressed under severe ABA treatment. It can be concluded that the three members were more sensitive to ABA treatment than the other members in potatoes. In Arabidopsis, *AtRCD1* participated in the signal transduction process of plant hormone ABA [11]. In this study, *AtRCD1*, *StSRO2* and *StSRO6* were distributed in group III with high homology, and *StSRO2* and *StSRO6* could actively respond to ABA stress. Therefore, this result fits well with previous studies.

The *SRO* gene family also responded to drought conditions under PEG stress treatment. In apples, *MdSRO2, MdSRO3* and *MdRCD1* were upregulated 18-fold, 17-fold and 14-fold, respectively. *MdRCD1* was supposed to be an essential regulator of abiotic stress, which regulated stomata through the ABA signal pathway and enhanced its tolerance to oxidative stress, drought and salt [13]. In Chinese cabbage, the genes responding to drought stress were *BrSRO1*, *BrSRO5* and *BrSRO9* [40]. In maize, the expression levels of *ZmSRO1e* and *ZmSRO1f* were upregulated considerably under drought stress [41]. In this study, the expression levels of *StSRO* genes in leaves were analyzed under PEG stress. The expression peak of *StSRO* genes was reached at the same time (12 h), and under severe drought stress (48 h), *StSRO2*, *StSRO3*, *StSRO5* and *StSRO6* were downregulated, while *StSRO1* and *StSRO4* did not respond to severe drought stress. 

Studies have suggested that the *SRO* gene family also plays a vital role in response to salt stress. In tomatoes, *SlSRO1* was significantly upregulated in roots under salt stress [38]. In wheat, *TaSRO2a.1*–*1D*, *TaSRO2a.2*–*4A* and *TaSRO2b.3*–*4A* were significantly upregulated in the root system under NaCl treatment. The above results indicated that these genes could quickly respond to salt stress [1]. In Chinese cabbage, the genes of *BrSRO5* and *BrSRO8* responded to NaCl stress [40]. In this study, the expression levels of *StSRO1* at different treatment times were significantly higher than the control. When the expression reached a peak, it was 18.9-fold that of the control, and *StSRO1*, *StSRO3* and *StSRO5* had the same expression pattern. This indicated that *StSROs* could respond positively to salt stress. In this study, it was found that *StSRO1*, *BrSRO5* and *BrSRO8* were distributed in group II by evolutionary analysis and showed high homology. At the same time, they could respond positively to salt stress. Therefore, the results of this study and previous studies are highly consistent. 

Studies have shown that different levels of salt stress will lead to varying degrees of molecular damage, growth arrest and even death of many salt-sensitive crops [49,50]. In upland cotton, two *SRO* genes, *GHSRO04* and *GHSRO08,* were cloned from upland cotton and expressed in cotton leaves under high salt stress (400 mM) and reached a peak at 24 h and 6 h, respectively, indicating that the *SRO* gene plays an important role in the salt stress of cotton [51]. In maize, the expression of *ZmSRO1a/ZmSRO1b/ZmSRO1c/ZmSRO1d/ZmSRO1e* was significantly upregulated in roots after 1 h of high salinity treatment. By comparison, the expression of *ZmSRO1f* in the shoots was upregulated considerably after high salinity treatment for 6 h [41]. In this research, the expression levels of *StSRO* genes were analyzed by RNA-seq data from the online database and qRT-PCR data under mild and moderate salt stress, and our transcriptome data under moderate and severe salt stress. The results showed that *StSRO3*, *StSRO5* and *StSRO6* were highly expressed under mild salt stress (6 h); the expression levels of *StSRO1* and *StSRO3*–*5* were higher under moderate salt stress (24 h); and *StSRO1* and *StSRO4*–*6* were highly expressed under severe salt stress for 48 h. Therefore, this study demonstrated that *StSRO* genes and *SRO* genes of other species played similar functions under adverse circumstances, and *StSRO* genes could respond to different salt conditions based on our transcriptome data. *StSRO* genes may be more inclined to respond to severe salt stress. *StSRO5* obviously alleviated mild, moderate and severe salt stress in potatoes and improved salt tolerance to a certain extent. 

## 4. Materials and Methods

### 4.1. Identification and Sequence Analysis of SRO Gene Family Members in Potato

To obtain the *StSRO* genes, the whole protein sequences of potatoes were retrieved and downloaded from the database of Spud DB Potato Genomics Resource (http://solanaceae.plantbiology.msu.edu/, accessed on 16 September 2022) [25]. The hidden Markov model files (HMM file) of the PARP, RST and WWE domains of *SRO* genes were downloaded from the online database Pfam (http://pfam.xfam.org/, accessed on 16 September 2022). Then, the Hmmsearch tool implemented in HMMER 3.0 was used to screen the ID of *StSRO* gene family members with the expected value (E-value) of 1e-5 [52,53]. Finally, the protein sequence of *StSRO* gene family members was extracted by TBtools 1.09876 and screened using the target domain by uploading the obtained protein sequence file to the online database pfamscan (https://www.ebi.ac.uk/Tools/pfa/pfamscan/, accessed on 16 September 2022) [54]. To ensure that the domains of the candidate genes contained at least one PARP and RST domain, the online website SMART (http://smart.embl-heidelberg.de/, accessed on 16 September 2022) was used for comparison with Blast-P of NCBI and verified the identified *StSRO* genes [55]. The physicochemical properties of StSRO proteins, including GRAVY, II, AI, pI, MW and the number of AA compositions, were obtained from ExPASY (https://web.expasy.org/compute_pi/, accessed on 16 September 2022) [56]. Subcellular localization of the *StSRO* genes was predicted by Wolf PSORT (https://wolfpsort.hgc.jp/, accessed on 16 September 2022) [57,58]. 

### 4.2. Gene Structure and Conserved Motifs of SRO Proteins in Potato

The conserved regions of StSROs protein were identified using the MEME program (https://meme-suite.org/meme/, accessed on 16 September 2022) [59]. The corresponding motif number parameter was set to 10, and the other parameters were set as default. The mast XML file was downloaded; then, the motif and gene structure of *StSRO* genes were visualized by TBtools.

### 4.3. Chromosomal Mapping and Secondary Structure Analysis of SRO Genes in Potato

To determine the distribution of *StSRO* genes on the chromosome, the chromosome location information of the identified *StSRO* genes was downloaded from the Spud DB Potato Genomics Resource database, and TBtools was used to draw the chromosome location map of *StSRO* genes. The secondary structure of StSRO proteins was predicted by the online SOPMA (https://npsa-prabi.ibcp.fr/cgi-bin/npsa_automat.pl?page=npsa SOPMA.html, accessed on 16 September 2022) [60].

### 4.4. Synteny Analysis of SRO Gene Family

MCScanX was used for gene duplication events in the potato genome and synteny relationship between potato and Arabidopsis, tomato. The collinearity relationship was visualized by TBtools, and default parameters were selected for all parameters [61].

### 4.5. Phylogenetic Tree Analysis of SRO Genes in Potato

The evolutionary relationships of *SRO* genes from four plant species, including potato, *Arabidopsis thaliana*, *Brassica pekinensis* and *Zea mays,* were investigated. The protein sequences of 6 *SRO* genes from *Arabidopsis thaliana*, 12 *SRO* genes from *Brassica pekinensis* and 6 *SRO* genes from *Zea mays* were downloaded from the database of Ensembl plants (https://plants.ensembl.org/index.html, accessed on 16 September 2022). Six *StSRO* gene protein sequence alignments were performed using ClustalW of MEGA-X, and the phylogenetic tree of the above four species was constructed by the neighbor-joining method. The repetition bootstrap was set to 1000, and all other parameters were set as default [62,63].

### 4.6. Analysis of Cis-Acting Elements of SRO Gene Family in Potato

The 2000 bp upstream sequence of the start codon of *StSRO* genes was extracted from the whole genome of potato by TBtools, and the cis-regulatory elements in the promoter region of *StSRO* genes were searched and analyzed by online plantcare (http://bioinformatics.psb.ugent.be/webtools/PlantCARE/html/, accessed on 16 September 2022) [64].

### 4.7. Construction of SRO Protein Interaction Network in Potato

The protein–protein interaction network of the *StSRO* gene family was predicted in potatoes based on the model plant Arabidopsis. The *SRO* genes in Arabidopsis were orthologous to those used in potatoes. The protein network interaction map was constructed using STRING (STRING: functional protein association networks (https://cn.string-db.org/, accessed on 16 September 2022) [65]. 

### 4.8. Gene Ontology (GO) Analysis of SRO Genes in Potato

Potato protein sequences were submitted to the website eggNOG-mapper (http://eggnog-mapper.embl.de/, accessed on 16 September 2022) for gene function classification and annotation, and the results of gene function annotation were further collated by TBtools [66].

### 4.9. Analysis of Potato SRO Gene Expression in Different Tissues and Different Treatments in DM Potato

The publicly available DM potato RNA sequencing data were downloaded from the database of Spud DB Potato Genomics resource. Then, the cluster heatmap was drawn using the plug-in heatmap of TBtools software.

### 4.10. RNA Isolation and qRT-PCR

The CDS sequence of *StSRO* genes was downloaded from the Spud DB Potato Genomics Resource database, and the primers were designed through NCBI (National Center for Biotechnology Information (https://www.ncbi.nlm.nih.gov/tools/primer-blast/, accessed on 16 September 2022). The sequences of all primers used for qRT-PCR analysis are listed in Supplementary Materials in Table S2. The elongation factor (*ef1α*) gene (Gen-Bank ID: AB061263) was used as the housekeeping gene to quantify cDNA abundance. Total RNA was extracted with Tiangen’s RNA easy fast plant tissue kit (DP452). Reverse transcription was performed using the cDNA Synthesis Kit (KR118) and determined by qRT-PCR. Three technical replicates were performed for each sample. The qRT-PCR data were used to calculate and analyze the relative expression of *StSRO* genes using the 2^−ΔΔCt^ quantitative method to determine differences between the treatments. The histogram was generated using the originPro 2018c software [67].

### 4.11. Plant Materials, Growth Conditions and Treatments

Potato cultivar “Zihuabai” was used as the material. Potato seedlings were propagated in vitro on liquid Murashige–Skoog (MS) medium and under a 16 h light/8 h dark cycle in an illuminated incubator maintained at 25 °C and 80% relative humidity (RH) and cultured for 30 d; then, plants with a similar growth trend were selected for treatment with the following stress treatments [68,69]. For the drought stress treatment, the original liquid MS medium was replaced with the new liquid MS medium containing 20% PEG-6000 and continued to be cultured under the same conditions as above. For the salt stress treatment, the original liquid medium was replaced with the new MS liquid medium containing 200 mM NaCl and then continued to be cultured under the same conditions as above. For the hormone ABA treatment, 100 μM ABA solution (freshly prepared) was evenly sprayed on the surface of plant leaves. The leaves were collected from plants at 0 h, 2 h, 6 h, 12 h, 24 h and 48 h after treatment. Samples immediately frozen at −80 °C were used for subsequent real-time fluorescence quantitative tests and transcriptome analysis. Transcriptome analysis was carried out on potato leaves treated with 200 mM NaCl for 24 h, 48 h and control (0 h). Leaf samples under each treatment were obtained with three independent biological replicates. 

### 4.12. RNA-Seq Data Analysis

Total RNA of the previous samples was extracted, and the RNA-seq library was constructed. Each sample had three biological replicates. Second-generation sequencing was executed by Gene Read Biotechnology (Wuhan, China). The raw RNA-seq data were not published. After RNA sequencing, the clean reads were obtained by removing the linker information, low-quality bases and undetected bases from the raw reads through fine filtering. The cleaned data were aligned to potato database DM v6.1 gene models downloaded from the potato database. 

### 4.13. Differential Expression Genes Analysis

Differential genes were identified using fragments per kilobase of exon per million fragments mapped (FPKM). Genes with the absolute value of |FC| ≥ 1.5 and *p*-value ≤ 0.05 were considered significantly differential expression genes (DEGs). The DEGs were annotated against the non-redundant database (Nr), SwissProt/UniProt Plant Proteins, Kyoto Encyclopedia of Genes and Genomes (KEGG) and eggNOG. Then, the DEGs were subjected to enrichment analysis of the KEGG pathways and GO functions. 

## 5. Conclusions

In this study, six *StSRO* genes were identified from the potato genome, and their corresponding structures and functions were analyzed. Analysis of the synteny relationships showed that only one pair of duplication genes was identified in the potato genome, which played a pivotal role in the expansion of the *StSRO* gene family. The results also indicated that two and seven pairs of *StSRO* genes were orthologous to Arabidopsis and tomato, respectively. The GO data showed that *StSRO* genes played an influential role in regulating gene expression, including genes in response to stress. *StSRO* genes were highly expressed in petiole and immature fruit. In addition, the leaves of potato seedlings wilted slightly under mild and moderate salt or drought stress, while they wilted and showed chlorosis under severe salt or drought stress, but they were more sensitive and intolerant of the ABA treatment. This study further analyzed the expression pattern of the *StSRO* gene under mild, moderate and severe drought stress, salt stress and ABA treatment by qRT-PCR. Furthermore, the expression profiling of *StSRO* genes in the “Zihuabai” potato cultivar under salt stress was analyzed by our transcriptome data under moderate and severe salt stress. The six *StSRO* genes displayed different expression patterns under different degrees of salt stress, which was consistent with the expression profiling of DM potato and qRT-PCR results. This study lays an important basis for further research on the function of the potato *SRO* gene family, especially the function of *StSRO* genes with potential functions under hormone induction and abiotic stress. It also provides an essential potential application value for stress-resistance breeding in potatoes.

## Figures and Tables

**Table 1:** *StSRO* genes in the PGSC database.

Gene Name	Gene ID (DM v6.1)	PARP	RST	WWE	Transcript ID (DM v6.1)	Transcript ID (DM v4.03/4.04)
*StSRO1*	Soltu.DM.03G028360	+	+	-	Soltu.DM.03G028360.1	PGSC0003DMT400063250
					Soltu.DM.03G028360.2	PGSC0003DMT400063249
					Soltu.DM.03G028360.3	PGSC0003DMT400063247
*StSRO2*	Soltu.DM.04G031810	+	+	-	Soltu.DM.04G031810.1	PGSC0003DMT400012724
*StSR* *O3*	Soltu.DM.05G008850	+	+	-	Soltu.DM.05G008850.1	PGSC0003DMT400035039
*StSRO4*	Soltu.DM.05G008950	+	+	-	Soltu.DM.05G008950.1	PGSC0003DMT400035057
					Soltu.DM.05G008950.2	
					Soltu.DM.05G008950.3	PGSC0003DMT400035056
*StSRO5*	Soltu.DM.06G018860	+	+	-	Soltu.DM.06G018860.1	PGSC0003DMT400043197
					Soltu.DM.06G018860.2	PGSC0003DMT400043196
*StSRO6*	Soltu.DM.08G022220	+	+	+	Soltu.DM.08G022220.1	PGSC0003DMT400038368

**Table 2:** Analysis of physical and chemical properties of *StSRO* gene family in potato.

Gene Name	Chr.	Protein Length (AA)	MW (kD)	pI	GRAVY	II	AI	SL
*StSRO1*	3	376	41,505.63	8.52	−0.446	49.19	68.46	N, C, CP, P
*StSRO2*	4	510	57,088.39	5.97	−0.234	38.53	87.82	CP, N, C, V, CY
*StSRO3*	5	319	33,719.99	9.16	−0.291	52.78	86.49	C, N, CP, E, CY
*StSRO4*	5	316	35,377.56	7.64	−0.260	47.20	88.20	C, N, E, V
*StSRO5*	6	594	67,469.25	6.55	−0.414	43.15	86.13	N, C, V
*StSRO6*	8	589	66,542.67	6.63	−0.431	46.06	81.19	N, C, CP, V

**Note:** AA. amino acid sequence length; MW. molecular weight; pI. isoelectric point; GRAVY. grand average of hydropathicity; II. instability index; AI. aliphatic index; SL. subcellular localization; N. nucleus; C. cytoplasm; CP. chloroplast; V. vacuole; P. peroxisome; E. extracellular matrix; CY. cytoskeleton.

**Table 3:** The KaKs values of the paired duplicated *StSRO* genes.

Duplicated Genes	Ka	Ks	Ka/Ks
*StSRO5*/*StSRO6*	0.26898	0.82258	0.32699

**Table 4:** The GO classification of the annotated *StSRO* genes in potato.

Class	GO Term	Annotation	StSRO Genes
MF	GO:0003824	catalytic activity	StSRO1, StSRO2, StSRO3, StSRO4, StSRO5, StSRO6
	GO:0005488	binding	StSRO1, StSRO5, StSRO6
	GO:0016740	transferase activity	StSRO1, StSRO2, StSRO3 StSRO4, StSRO5, StSRO6
	GO:0005515	protein binding	StSRO1, StSRO5, StSRO6
CC	GO:0005634	nucleus	StSRO1, StSRO2, StSRO3, StSRO4, StSRO5, StSRO6
	GO:0110165	cellular anatomical entity	StSRO1, StSRO2, StSRO3, StSRO4, StSRO5, StSRO6
	GO:0005622	intracellular anatomical structure	StSRO1, StSRO2, StSRO3, StSRO4, StSRO5, StSRO6
	GO:0005737	cytoplasm	StSRO1, StSRO5, StSRO6
	GO:0043226	organelle	StSRO1, StSRO2, StSRO3, StSRO4, StSRO5, StSRO6
	GO:0043227	membrane-bounded organelle	StSRO1, StSRO2, StSRO3, StSRO4, StSRO5, StSRO6
	GO:0005739	mitochondrion	StSRO1
	GO:0043229	intracellular organelle	StSRO1, StSRO2, StSRO3, StSRO4, StSRO5, StSRO6
	GO:0043231	intracellular membrane-bounded organelle	StSRO1, StSRO2, StSRO3, StSRO4, StSRO5, StSRO6
BP	GO:0009987	cellular process	StSRO1, StSRO5, StSRO6
	GO:0000003	reproduction	StSRO2, StSRO5, StSRO6
	GO:0051716	cellular response to stimulus	StSRO5, StSRO6
	GO:0009605	response to external stimulus	StSRO5, StSRO6
	GO:0009607	response to biotic stimulus	StSRO5, StSRO6
	GO:0009416	response to light stimulus	StSRO2
	GO:0023052	signaling	StSRO5, StSRO6
	GO:0050896	response to stimulus	StSRO1, StSRO2, StSRO5, StSRO6
	GO:0008152	metabolic process	StSRO1, StSRO5, StSRO6
	GO:0048856	anatomical structure development	StSRO2, StSRO5, StSRO6
	GO:0008219	cell death	StSRO5, StSRO6
	GO:0009628	response to abiotic stimulus	StSRO1, StSRO2, StSRO5, StSRO6
	GO:0009058	biosynthetic process	StSRO5, StSRO6
	GO:0009314	response to radiation	StSRO2
	GO:0050789	regulation of biological process	StSRO5, StSRO6
	GO:0006950	response to stress	StSRO1, StSRO2, StSRO5, StSRO6
	GO:0050794	regulation of cellular process	StSRO5, StSRO6
	GO:0007275	multicellular organism development	StSRO2, StSRO5, StSRO6
	GO:0042221	response to chemical	StSRO2, StSRO5, StSRO6
	GO:0065007	biological regulation	StSRO5, StSRO6
	GO:0007154	cell communication	StSRO5, StSRO6
	GO:0032501	multicellular organismal process	StSRO2, StSRO5, StSRO6
	GO:0032502	developmental process	StSRO2, StSRO5, StSRO6
	GO:0009719	response to endogenous stimulus	StSRO5, StSRO6
	GO:0007165	signal transduction	StSRO5, StSRO6
	GO:0009790	embryo development	StSRO2, StSRO5, StSRO6
	GO:0009791	post-embryonic development	StSRO2, StSRO5, StSRO6

## Data Availability

Not applicable.

## Supplementary Materials

The following supporting information can be downloaded at: https://www.mdpi.com/article/10.3390/ijms232113518/s1.
